# Changes in breaststroke swimming performances in national and international athletes competing between 1994 and 2011 –a comparison with freestyle swimming performances

**DOI:** 10.1186/2052-1847-6-18

**Published:** 2014-05-09

**Authors:** Mathias Wolfrum, Christoph Alexander Rüst, Thomas Rosemann, Romuald Lepers, Beat Knechtle

**Affiliations:** 1 Institute of General Practice and for Health Services Research, University of Zurich, Zurich, Switzerland; 2 Cardiovascular Center Cardiology, University Hospital Zürich, Zürich, Switzerland; 3 INSERM U1093, Faculty of Sport Sciences, University of Burgundy, Dijon, France; 4 Gesundheitszentrum St. Gallen, Vadianstrasse 26, 9001 St. Gallen, Switzerland

**Keywords:** Swimming speed, Sex-related difference, Gender difference, Men, Women

## Abstract

**Background:**

The purpose of the present study was to analyse potential changes in performance of elite breaststroke swimmers competing at national and international level and to compare to elite freestyle swimming performance.

**Methods:**

Temporal trends in performance of elite breaststroke swimmers were analysed from records of the Swiss Swimming Federation and the FINA (Fédération Internationale de Natation) World Swimming Championships during the 1994–2011 period. Swimming speeds of elite female and male breaststroke swimmers competing in 50 m, 100 m, and 200 m were examined using linear regression, non-linear regression and analysis of variance. Results of breaststroke swimmers were compared to results of freestyle swimmers.

**Results:**

Swimming speed in both strokes improved significantly (*p* < 0.0001-0.025) over time for both sexes, with the exception of 50 m breaststroke for FINA men. Sex differences in swimming speed increased significantly over time for Swiss freestyle swimmers (*p* < 0.0001), but not for FINA swimmers for freestyle, while the sex difference remained stable for Swiss and FINA breaststroke swimmers. The sex differences in swimming speed decreased significantly (*p* < 0.0001) with increasing race distance.

**Conclusions:**

The present study showed that elite male and female swimmers competing during the 1994–2011 period at national and international level improved their swimming speed in both breaststroke and freestyle. The sex difference in freestyle swimming speed consistently increased in athletes competing at national level, whereas it remained unchanged in athletes competing at international level. Future studies should investigate temporal trends for recent time in other strokes, to determine whether this improvement is a generalized phenomenon.

## Background

Improved understanding of performance by top athletes, including long-term changes and sex-related differences, can help athletes and coaches to estimate performance limits, to choose appropriate training protocols, and to set realistic goals. During the past 30 years, several studies investigated human limits in various sports such as running [1-3], track and field [4], tennis [4], and swimming [3]. However, the results were inconsistent. For example, Whipp and Ward [5] predicted that there would be no limits to human performance in running, and women would eventually run faster than men, while Cheuvront *et al.*[2] concluded that running performances by both men and women had already reached a plateau. Similarly, Nevill *et al.*[3] suggested that freestyle swimming performance had reached the limits of human capability. World record speeds improved significantly during the 1960s and 1970s, but levelled off early in the 21^st^ century [3]. This conclusion was further supported by Seiler *et al.*[6] and Johnson *et al.*[7]. However, temporal trends have not been examined in other strokes, and in fact, new world records in breaststroke and freestyle swimming were set by both men and women during the 2012 Olympic Games [8], suggesting that swimming performance has not reached its limit.

For swimmers, changes in performance over time have mainly been investigated in freestyle swimming [3,9-11]. Nevill *et al.* reported that the 10% faster performance in men compared to women in various freestyle swimming events remained unchanged during the last 60 years [3]. However, it is not known whether or not this is also true for breaststroke swimming. Differences between freestyle and breaststroke do exist in terms of technique, energy cost and stroke length [12]. It has been shown that freestyle is the most economic stroke, followed by backstroke, butterfly and breaststroke [13].

The changes in swimming performance have been investigated for freestyle swimmers competing at international top level [3,9,10]. Little data is known for changes in freestyle swimming performance across years for swimmers competing at national level [11] and no data exist for changes in swimming performance over time for other strokes such as breaststroke [14]. Differences in performance between athletes competing at international and national level are related to differences in the energetic and biomechanical profiles of these athletes [15-18]. Considering breaststroke, Seifert *et al.*[19] reported differences in the elbow-knee continuous relative phase in breaststroke swimmers of different performance levels. Furthermore, recent studies reported greater sex-related differences in swimming speed for freestyle than for breaststroke for swimmers at national level, but not for swimmers competing at international level [14]. This finding might be attributed to the biomechanics of the two swimming styles [20,21]. However, there is now comprehensive data about the temporal trend over recent years for the sex differences in breaststroke and freestyle swimming.

The purpose of the present study was to investigate the changes in breaststroke swimming performance in athletes competing at both national and international level and to compare breaststroke swimming performance to freestyle swimming performance. We therefore analyzed changes in freestyle and breaststroke performance of the annual top ten Swiss swimmers (*i.e.* national level) and the eight finalists in the Fédération Internationale de Natation (FINA) World Championships (*i.e.* international level) during the 1994–2011 period for 50 m, 100 m, and 200 m. The aims of the present study were to investigate (*i*) potential changes in breaststroke swimming performance across years in national and international athletes and to compare to potential changes in freestyle swimming performance, (*ii*) differences in swimming performance between national and international athletes and (*iii*) differences in swimming performance between women and men. We hypothesized that (*i*) performance in breaststroke swimmers would improve over time, (*ii*) national athletes would compete slower that international athletes and (*iii*) men would be faster than women for all distances with a constant sex difference in swimming performance.

## Methods

### Ethics

All procedures used in the study were approved by the Institutional Review Board of Kanton St. Gallen, Switzerland with a waiver of the requirement for informed consent of the participants given the fact that the study involved the analysis of publicly available data.

### Subjects and design

Race times on long courses for the annual top ten men and women in breaststroke and freestyle swimming recorded in the Swiss high score list between 1994 and 2011 were obtained per civil year from the website of the Swiss Swimming Federation [22]. The Swiss Swimming Federation records only the annual best swimming performance for each athlete, so no Swiss athlete was included more than once in the same year. Race times for the eight female and male finalists competing on international level in breaststroke and freestyle in the FINA (Fédération Internationale de Natation) World Swimming Championships between 1994 and 2011 were obtained from the website of the FINA [23]. Race times for athletes competing on national level were available annually for swimmers for both breaststroke and freestyle and all distances (*i.e.* 50 m, 100 m, and 200 m). For FINA finalists, race times were available from the World Championships in Rome (1994), Perth (1998) Fukuoka (2001), Barcelona (2003), Montreal (2005), Melbourne (2007), Rome (2009) and Shanghai (2011). The 50 m breaststroke was held for the first time in the 2001 World Championships.

### Methodology

Since races in breaststroke swimming were held for 50 m, 100 m and 200 m but freestyle races for 50 m, 100 m, 200 m, 400 m, 800 m and 1,500 m, we analysed freestyle swimming races only for 50 m, 100 m, and 200 m to compare with breaststroke swimming results. To allow a comparison of swimming performance for different styles and distances, race times were transformed to swimming speed by dividing race distance by time. To determine temporal trends, we compared the average annual swimming speed for each stroke and race distance, by the top ten Swiss men and top ten Swiss women, and by the eight men and eight women competing in the FINA finals. To analyse the maximum overall swimming performance, we averaged the fastest ten swimming speeds in each stroke for four groups: Swiss women, Swiss men, FINA women, and FINA men. Sex-related differences were calculated using the equation [(women swimming speed) – (men swimming speed)]/(men swimming speed) × 100. The calculation was performed for pairs of equally placed athletes during each year, *e.g.,* the fastest women and men, the second fastest women and men, etc*.* The absolute value of the sex-related difference for each pair was used to calculate the annual mean and standard deviation.

### Statistical analyses

Prior to statistical analyses, each data set was tested for normal distribution and homogeneity of variances. Normal distribution was tested using a D’Agostino and Pearson omnibus normality test. Homogeneity of variances was tested using a Levene’s test, in cases with two groups, and with a Bartlett’s test, in cases with more than two groups. A potential change in swimming speed across years was investigated using regression analyses. Since the change in sex difference in endurance is assumed to be non-linear [24], we additionally calculated the non-linear regression model that fits the data best. For swimmers at national level, polynomial regressions from 2^nd^ to 17^th^ degree were calculated; for swimmers at international level, polynomial regressions from 2^nd^ to 7^th^ degree were calculated. Additionally, LOWESS (*i.e.* locally weighted scatterplot smoothing) and 64 further standard models were used. We compared the best-fit non-linear models to the linear models using Akaike’s Information Criteria (AIC) and F-test in order to show which model (*i.e.* linear versus non-linear) would be the most appropriate to explain the trend of the data. One-way analysis of variance (ANOVA) with subsequent Tukey-Kramer post-hoc tests were used to compare data for multiple groups. A two-way ANOVA with a Bonferroni post-hoc test was used to determine the significance of interactive effects of swimming style and sex on performance. Significance of all statistical tests was accepted at *p* < 0.05. Statistical analyses were performed using IBM SPSS Statistics (Version 19 and 20, IBM SPSS, Chicago, IL, USA) and GraphPad Prism (Version 5 and 6.01, GraphPad Software, La Jolla, CA, USA). Data are reported in the text and figures as mean ± standard deviation (SD).

## Results

### Changes in breaststroke and freestyle swimming speed across the years

**Figure 1:**
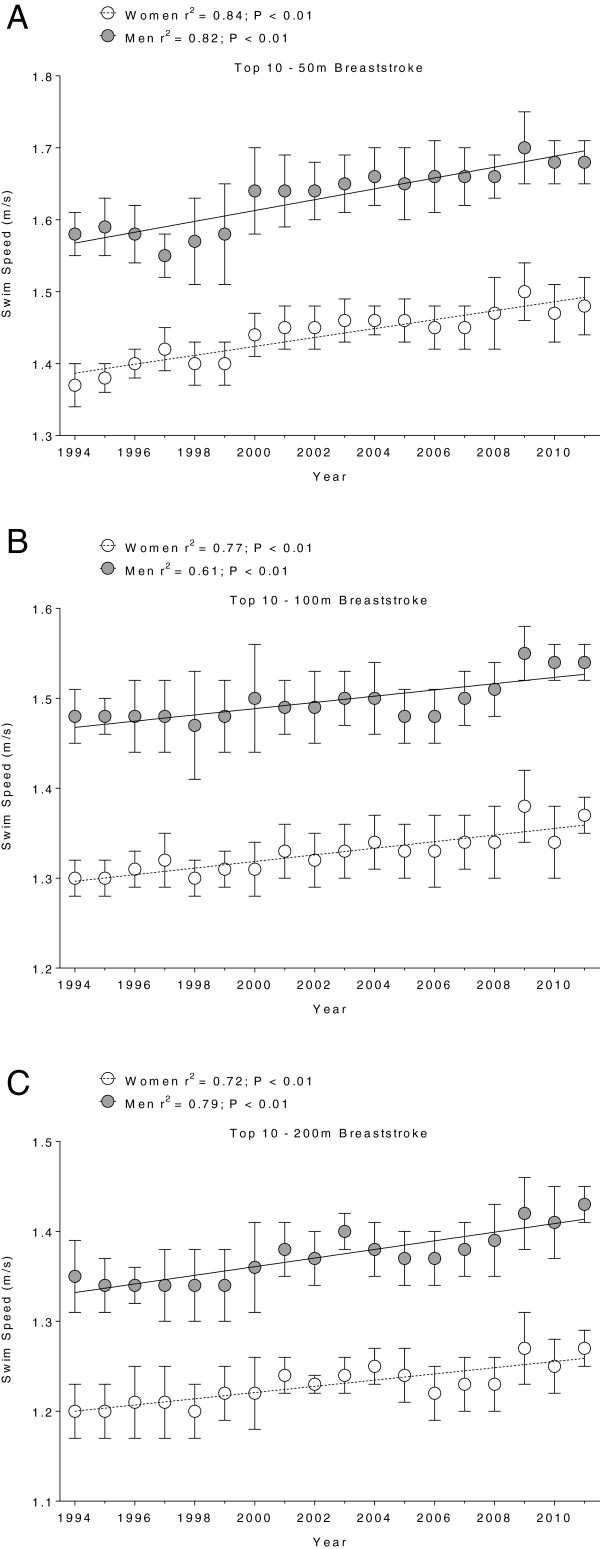
**Swimming speeds of Swiss top ten breaststroke swimmers for 50 m (Panel A), 100 m (Panel B) and 200 m (Panel C).** Results are presented as mean ± SD.

**Figure 2:**
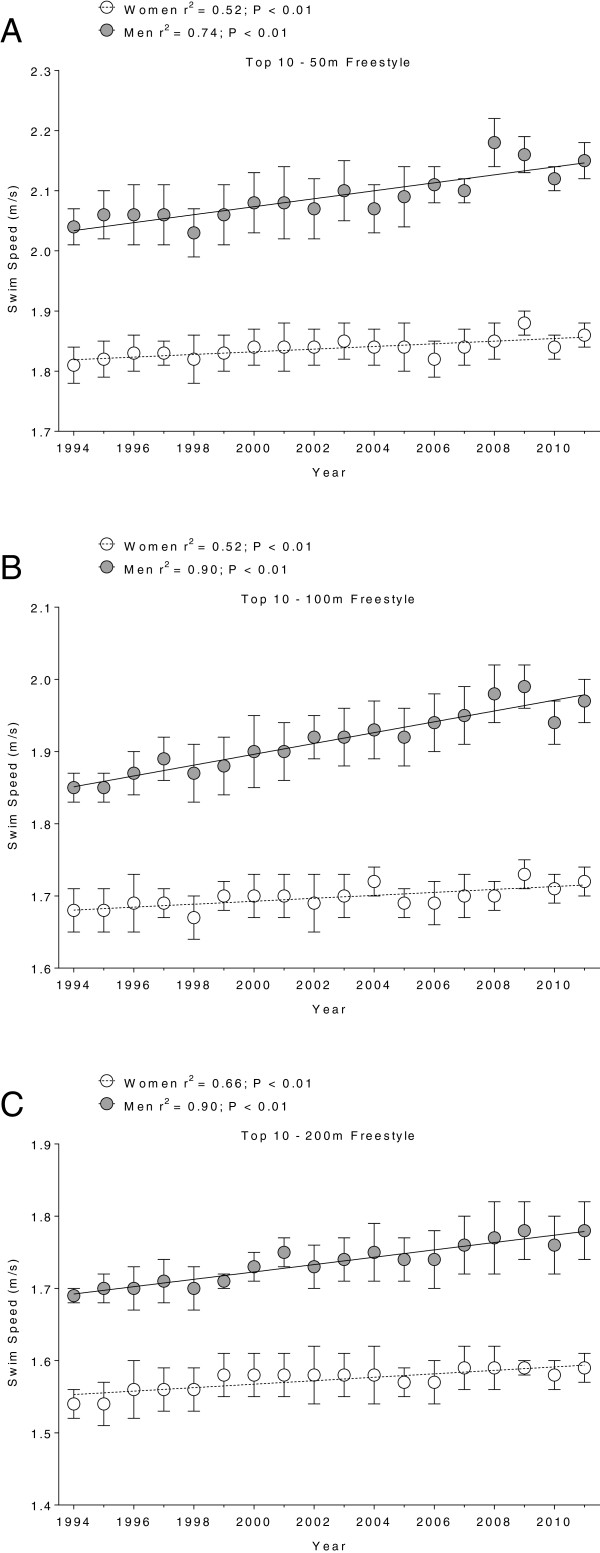
**Swimming speeds of Swiss top ten freestyle swimmers for 50 m (Panel A), 100 m (Panel B) and 200 m (Panel C).** Results are presented as mean ± SD.

**Figure 3:**
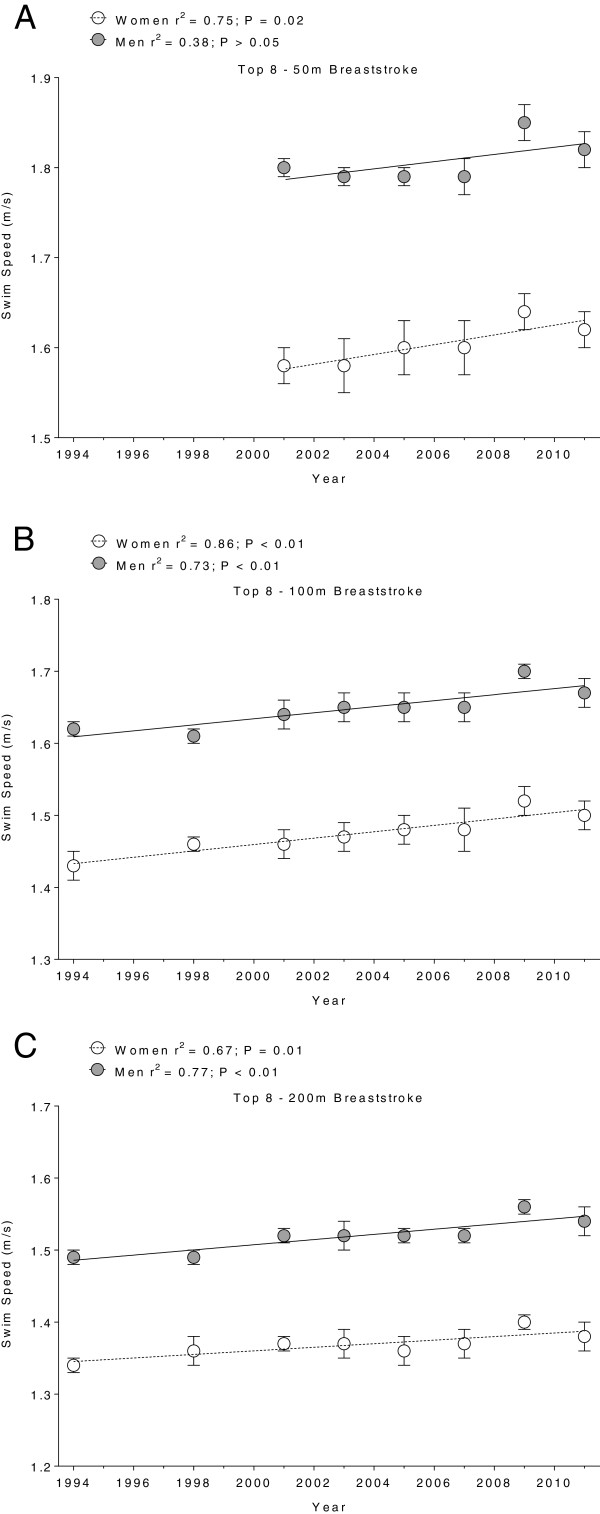
**Swimming speeds of breaststroke FINA finalists for 50 m (Panel A), 100 m (Panel B) and 200 m (Panel C).** Results are presented as mean ± SD.

**Figure 4:**
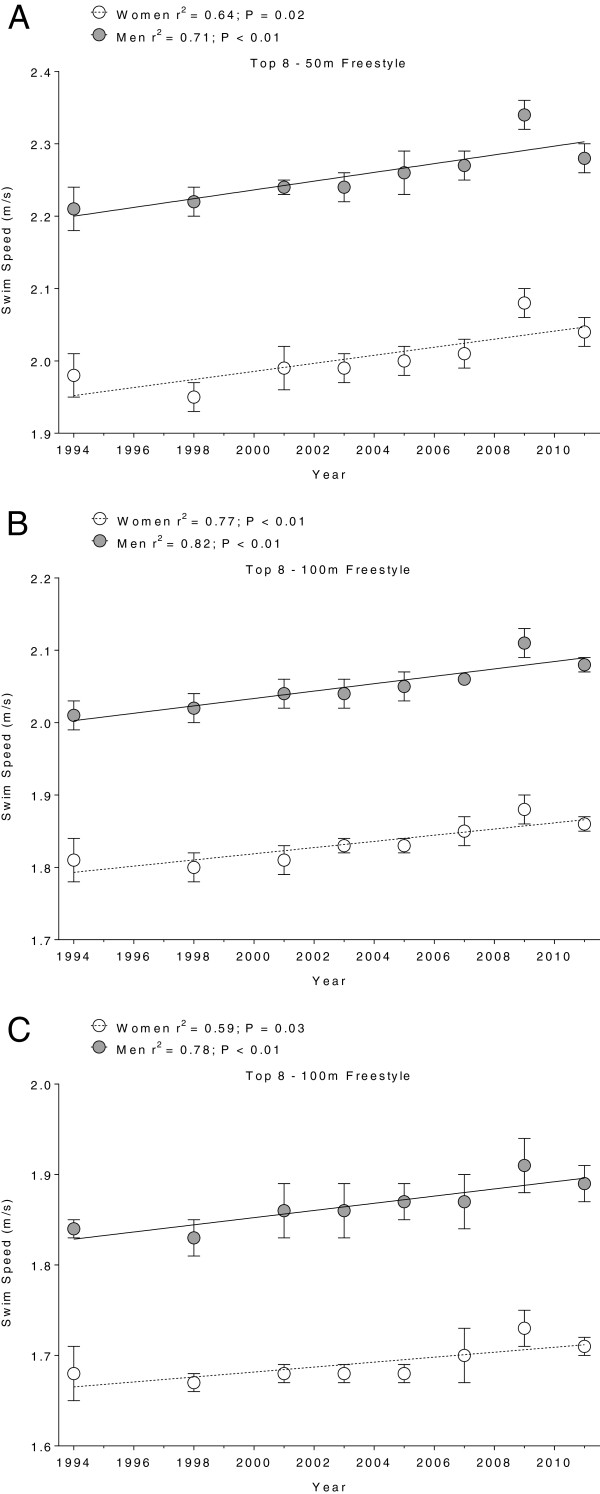
**Swimming speeds of freestyle FINA finalists for 50 m (Panel A), 100 m (Panel B) and 200 m (Panel C).** Results are presented as mean ± SD.

Figure 1,2,3 and 4 present the changes in swimming speeds for breaststroke (Figure 1) and freestyle (Figure 2) in Swiss swimmers and for breaststroke (Figure 3) and freestyle (Figure 4) in FINA swimmers for 50 m (Panels A), 100 m (Panels B) and 200 m (Panels C). Swimming speed increased linearly for Swiss swimmers in breaststroke (Table 1) and freestyle (Table 2). In FINA finalists, swimming speed increased also linearly in breaststroke (Table 3) and freestyle (Table 4), with the exception of male FINA swimmers in the 50 m breaststroke (Figure 3A).

**Table 1:** Comparison of linear and non-linear regression analysis of changes in swimming speed for female and male breast stroke swimmers at national level across years to determine which model is the best

	**Kind of regression**	**Sum of Squares**	**DOF**	**AICC**	**Best regression AIC-Test**	**Best regression F-Test**	**Delta**	**Probability**	**Likelihood**
50 m breaststroke	polynomial	0.0035	12	−138.34	linear	linear	2.80	0.19	80.3%
male Swiss swimmers	linear	0.0062	16	−141.15					
100 m breaststroke	polynomial	0.0009	0	−144.16	linear	undetermined	5.72	0.054	94.6%
male Swiss swimmers	linear	0.0038	16	−149.88					
200 m breaststroke	polynomial	0.0011	0	−139.69	linear	undetermined	13.48	0.0011	99.88%
male Swiss swimmers	linear	0.0032	16	−153.17					
50 m breaststroke	polynomial	0.0011	0	−140.12	linear	undetermined	6.62	0.035	96.5%
female Swiss swimmers	linear	0.0045	16	−146.74					
100 m breaststroke	polynomial	0.0018	0	−131.76	linear	undetermined	29.91	3.19 e^−07^	100%
female Swiss swimmers	linear	0.0019	16	−161.67					
200 m breaststroke	polynomial	0.0014	0	−135.81	linear	undetermined	22.41	1.35 e^-05^	99.99%
female Swiss swimmers	linear	0.0024	16	−158.22					

For all distances, the changes in swimming speed were linear.

**Table 2:** Comparison of linear and non-linear regression analysis of changes in swimming speed for female and male freestyle swimmers at national level across years to determine which model is the best

	**Kind of regression**	**Sum of Squares**	**DOF**	**AICC**	**Best regression AIC-Test**	**Best regression F-Test**	**Delta**	**Probability**	**Likelihood**
50 m freestyle	polynomial	0.0067	0	−108.11	linear	undetermined	29.06	4.87 e^-07^	100%
male Swiss swimmers	linear	0.0077	16	−137.18					
100 m freestyle	polynomial	0.0002	2	−45.86	linear	linear	21.05	2.67 e^-05^	99.99%
male Swiss swimmers	linear	0.0013	6	−66.92					
200 m freestyle	polynomial	0.0015	0	−134.90	linear	linear	32.22	1.005 e^-07^	100%
male Swiss swimmers	linear	0.0014	16	−167.13					
50 m freestyle	polynomial	0.0016	0	−132.88	linear	undetermined	27.20	1.23 e^-06^	99.99%
female Swiss swimmers	linear	0.0021	16	−160.08					
100 m freestyle	polynomial	0.0027	0	−124.34	linear	undetermined	28.91	5.26 e^-07^	99.99%
female Swiss swimmers	linear	0.0031	16	−153.25					
200 m freestyle	polynomial	0.0006	0	−152.27	linear	undetermined	14.68	0.00064	99.93%
female Swiss swimmers	linear	0.0014	16	−166.95					

For all distances, the changes were linear.

**Table 3:** Comparison of linear and non-linear regression analysis of changes in swimming speed for female and male breast stroke swimmers at international level across years to determine which model is the best

	**Kind of regression**	**Sum of Squares**	**DOF**	**AICC**	**Best regression AIC-Test**	**Best regression F-Test**	**Delta**	**Probability**	**Likelihood**
50 m breaststroke	polynomial	0.00042	1	−9.35	linear	linear	35.83	1.65 e^-08^	100%
male FINA finalists	linear	0.00190	4	−45.19					
100 m breaststroke	polynomial	0.00035	2	−40.28	linear	linear	23.83	6.66 e^-06^	99.99%
male FINA finalists	linear	0.00180	6	−64.12					
200 m breaststroke	polynomial	0.00014	2	−47.16	linear	linear	23.19	9.18 e^-06^	99.99%
male FINA finalists	linear	0.00086	6	−70.35					
50 m breaststroke	polynomial	0.00027	0	−49.89	linear	undetermined	1.51	0.31	68.04%
female FINA finalists	linear	0.00069	4	−51.41					
100 m breaststroke	polynomial	0.00034	2	−40.31	linear	linear	30.66	2.19 e^-07^	100%
female FINA finalists	linear	0.00080	6	−70.97					
200 m breaststroke	polynomial	8.76 e^-05^	2	−51.37	linear	linear	18.04	0.00012	99.98%
female FINA finalists	linear	0.00097	6	−69.41					

For all distances, the changes were linear.

**Table 4:** Comparison of linear and non-linear regression analysis of changes in swimming speed for female and male freestyle swimmers at international level across years to determine which model is the best

	**Kind of regression**	**Sum of Squares**	**DOF**	**AICC**	**Best regression AIC-Test**	**Best regression F-Test**	**Delta**	**Probability**	**Likelihood**
50 m freestyle	polynomial	0.00061	2	−35.82	linear	linear	23.53	7.76 e^-06^	99.99%
male FINA finalists	linear	0.00340	6	−59.35					
100 m freestyle	polynomial	0.00017	2	−45.86	linear	linear	32.22	1.005 e^−07^	100%
male FINA finalists	linear	0.00130	6	−66.92					
200 m freestyle	polynomial	0.00020	2	−44.73	linear	linear	23.55	7.69 e^-06^	99.99%
male FINA finalists	linear	0.00110	6	−68.28					
50 m freestyle	polynomial	0.00039	2	−39.26	linear	linear	18.46	9.76 e^-05^	99.99%
female FINA finalists	linear	0.00420	6	−57.73					
100 m freestyle	polynomial	0.00019	2	−44.78	linear	linear	22.05	1.62 e^-05^	99.99%
female FINA finalists	linear	0.00130	6	−66.84					
200 m freestyle	polynomial	1.58 e^-05^	2	−65.03	linear	polynomial	1.24	0.34	65.08%
female FINA finalists	linear	0.00140	6	−66.27					

The changes were linear for all distances.

In breaststroke, male Swiss swimmers increased their swimming speed by 0.008 m · s^−1^, 0.004 m · s^−1^, and 0.005 m · s^−1^*per annum* in 50 m, 100 m, and 200 m, respectively, and female swimmers by 0.006 m · s^−1^, 0.004 m · s^−1^, 0.004 m · s^−1^*per annum*, respectively*.* Male FINA swimmers increased swimming speed by 0.004 m · s^−1^*per annum* over 100 m and 200 m, respectively, and female swimmers by 0.005 m · s^−1^, 0.004 m · s^−1^, 0.003 m · s^−1^*per annum* in 50 m, 100 m, and 200 m, respectively. In freestyle, male Swiss swimmers increased swimming speed by 0.007 m · s^−1^, 0.008 m · s^−1^, and 0.005 m · s^−1^*per annum* in 50 m, 100 m, and 200 m, respectively, and female swimmers by 0.002 m · s^−1^*per annum* for all distances. Male FINA swimmers increased swimming speed by 0.006 m · s^−1^, 0.005 m · s^−1^, and 0.004 m · s^−1^*per annum* in 50 m, 100 m, and 200 m, respectively, and female swimmers by 0.006 m · s^−1^, 0.004 m · s^−1^, and 0.003 m · s^−1^*per annum*, respectively.

### Sex differences in breaststroke and freestyle swimming speed

**Figure 5:**
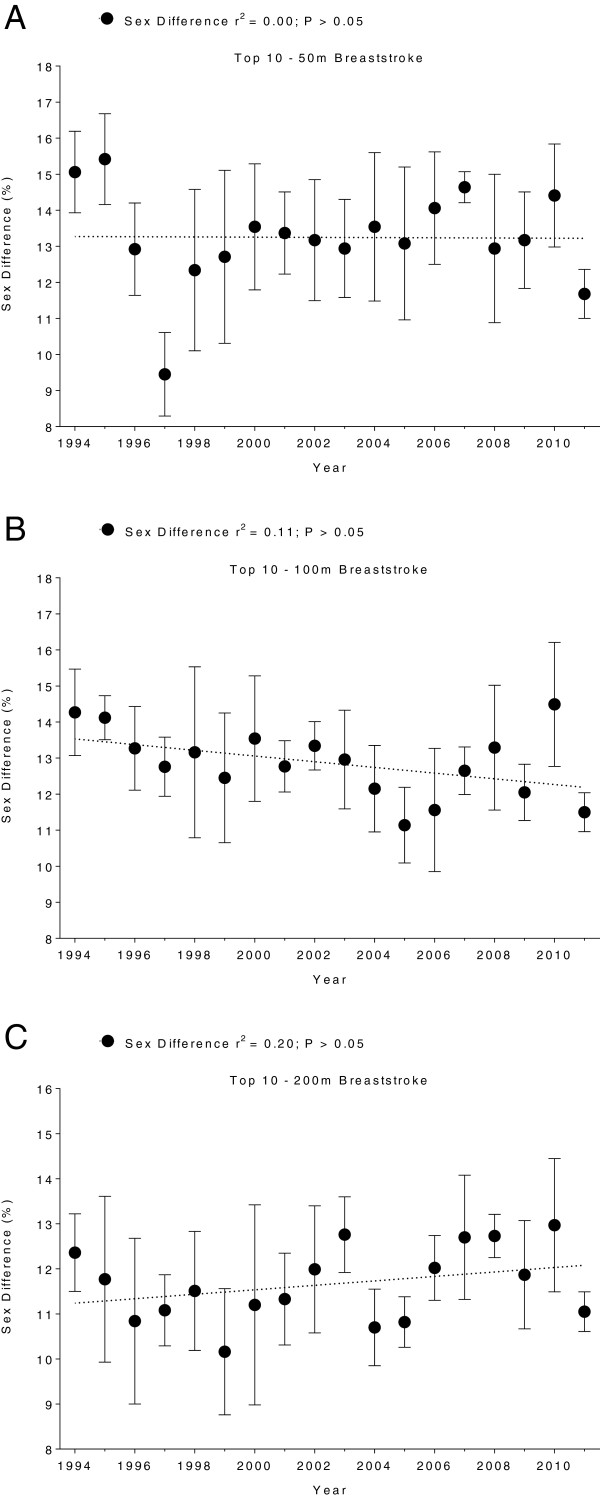
**Sex differences of Swiss top ten breaststroke swimmers for 50 m (Panel A), 100 m (Panel B) and 200 m (Panel C).** Results are presented as mean ± SD.

**Figure 6:**
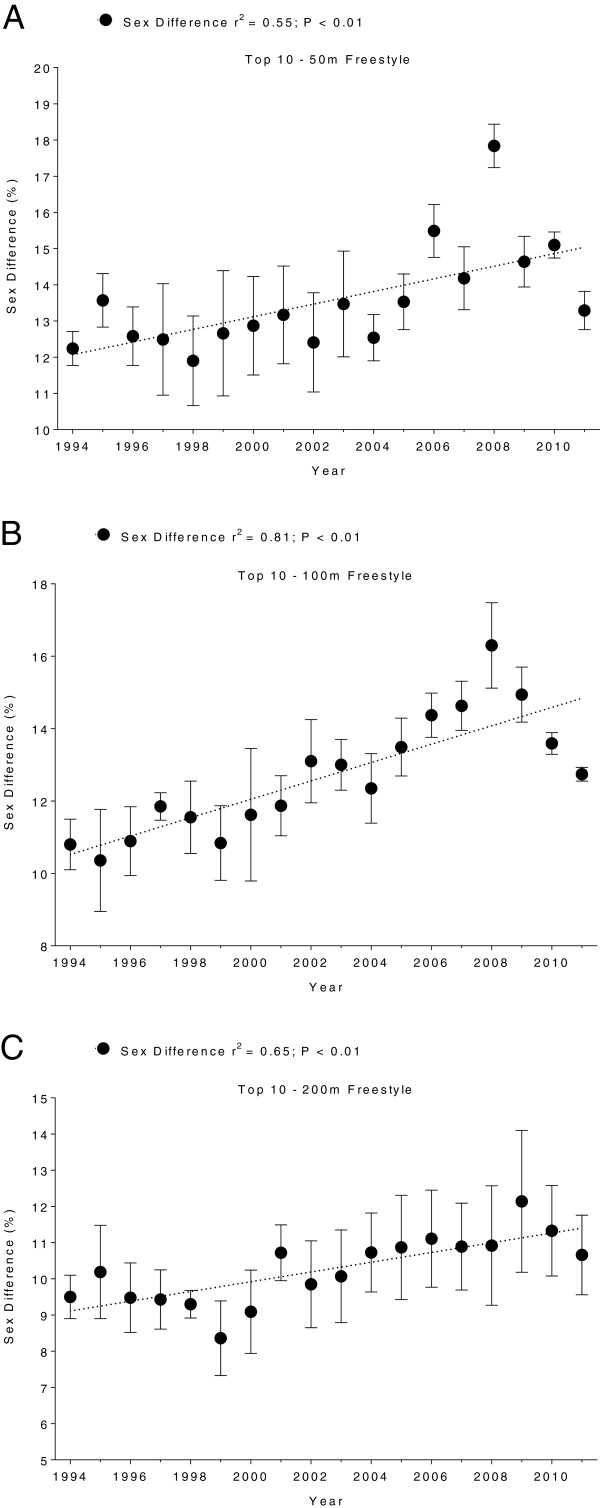
**Sex differences of Swiss top ten freestyle swimmers for 50 m (Panel A), 100 m (Panel B) and 200 m (Panel C).** Results are presented as mean ± SD.

**Figure 7:**
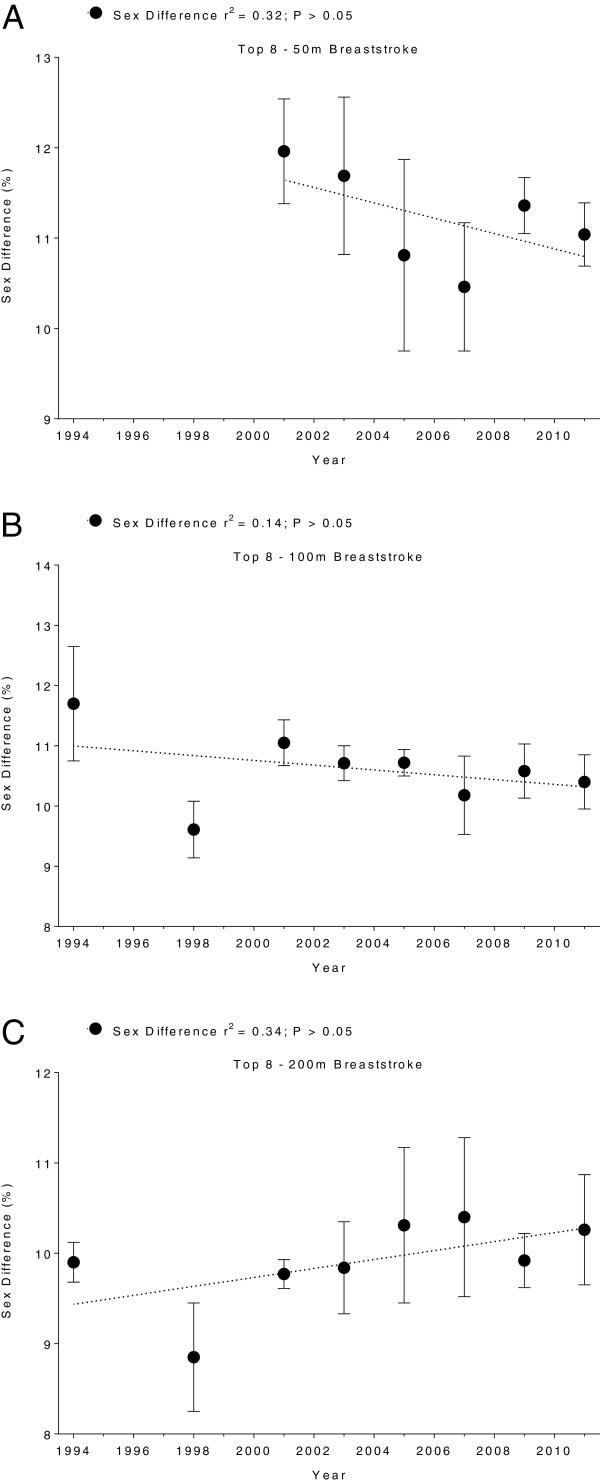
**Sex differences of breaststroke FINA finalists for 50 m (Panel A), 100 m (Panel B) and 200 m (Panel C).** Results are presented as mean ± SD.

**Figure 8:**
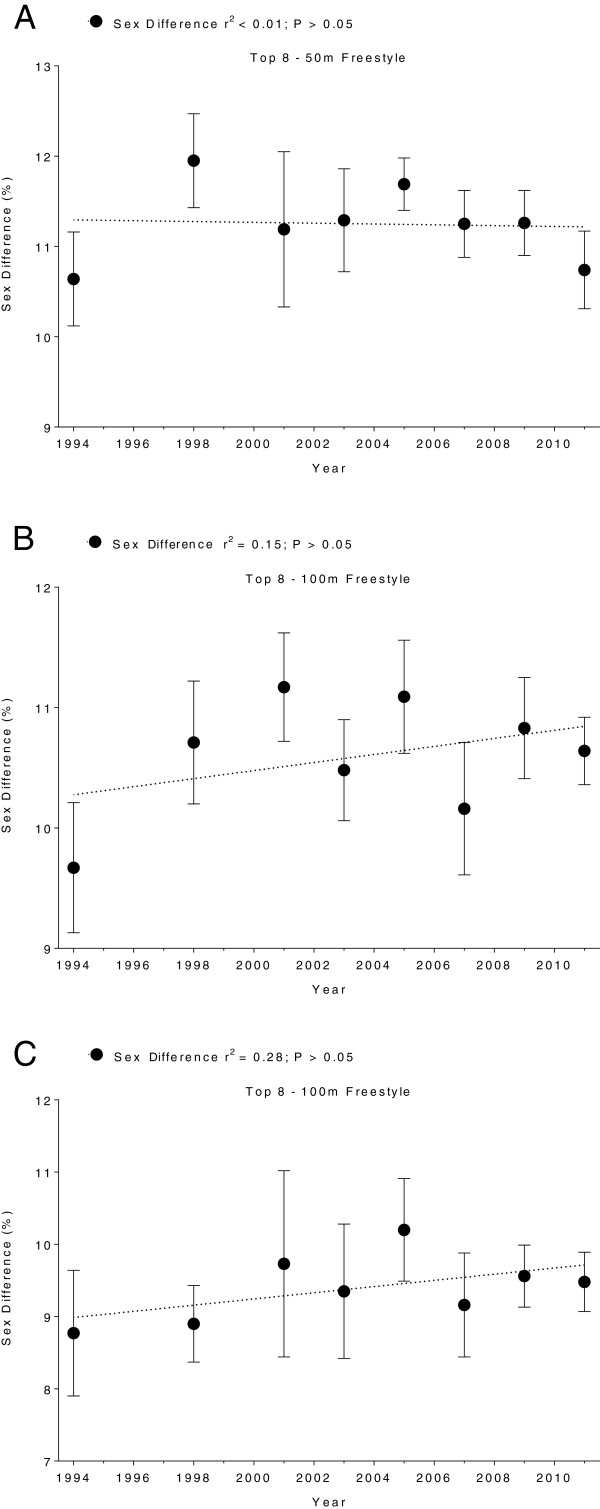
**Sex differences of freestyle FINA finalists for 50 m (Panel A), 100 m (Panel B) and 200 m (Panel C).** Results are presented as mean ± SD.

**Figure 9:**
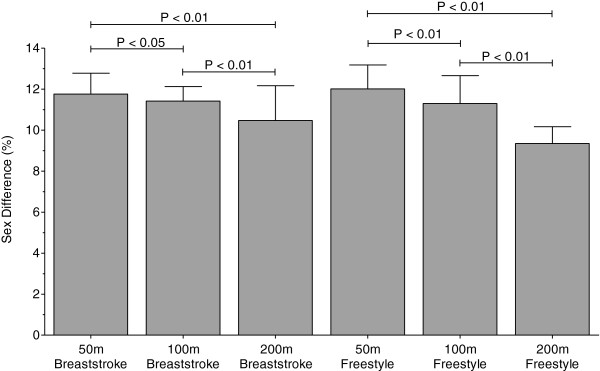
**Comparison of sex differences in swimming speeds of the top ten Swiss breaststroke and freestyle swimmers for 50 m, 100 m and 200 m.** Results are presented as mean ± SD.

**Figure 10:**
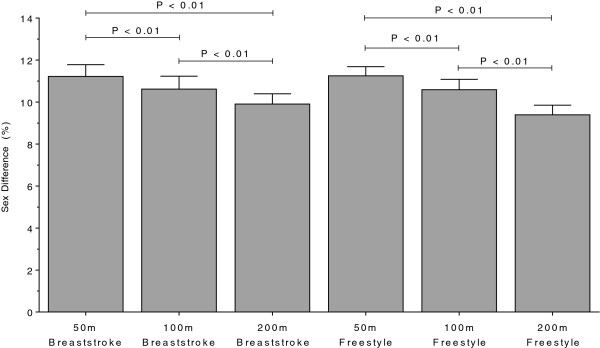
**Comparison of sex differences in swimming speeds of FINA finalists in breaststroke and freestyle for 50 m, 100 m and 200 m.** Results are presented as mean ± SD.

Men swam consistently faster than women for both strokes and all distances. In Swiss swimmers, swimming speed in breaststroke showed no changes across years (Figure 5 and Table 5). In freestyle, however, the sex difference in swimming speed increased linearly (Table 6) for all distances (Figure 6). In FINA finalists, the sex differences in swimming speed showed no significant trends over time (Table 6) for breaststroke (Figure 7) and for freestyle (Figure 8). Sex differences in both breaststroke and freestyle performance decreased significantly (*p* < 0.0001) with increasing race distance for both Swiss (Figure 9) and FINA swimmers (Figure 10) (*i.e.* for breaststroke at national level 50 m 11.8%, 100 m 11.4% and 200 m 10.5%; international level 50 m 11.2%, 100 m 10.6% and 200 m 9.9%; for freestyle at national level 50 m 12.0%, 100 m 11.3% and 200 m 9.3%, international level 50 m 11.3%, 100 m 10.6%, 200 m 9.4%, respectively).

**Table 5:** Comparison of linear and non-linear regression analysis of changes in sex difference for freestyle and breaststroke swimmers at national level across years to determine which model is the best

	**Kind of regression**	**Sum of Squares**	**DOF**	**AICC**	**Best regression AIC-Test**	**Best regression F-Test**	**Delta**	**Probability**	**Likelihood**
50 m breaststroke	polynomial	10.02	0	23.45	linear	undetermined	21.71	1.92 e^-5^	99.99%
Swiss swimmers	linear	17.49	16	1.73					
100 m breaststroke	polynomial	2.78	0	0.40	linear	linear	13.75	0.0010	99.89%
Swiss swimmers	linear	7.56	16	−13.35					
200 m breaststroke	polynomial	3.80	0	6.03	linear	undetermined	22.42	1.34 e^-5^	99.99%
Swiss swimmers	linear	6.38	18	−16.39					
50 m freestyle	polynomial	7.20	0	17.51	linear	undetermined	24.94	3.83 e^-6^	99.99%
Swiss swimmers	linear	10.51	16	−7.42					
100 m freestyle	polynomial	3.93	13	−16.30	linear	linear	1.30	0.34	65.7%
Swiss swimmers	linear	5.97	16	−17.61					
200 m freestyle	polynomial	1.90	0	−6.42	linear	undetermined	18.32	0.00010	99.98%
Swiss swimmers	linear	4.01	16	−24.74					

The changes were linear for all distances.

**Table 6:** Comparison of linear and non-linear regression analysis of changes in sex difference for freestyle and breaststroke swimmers at international level across years to determine which model is the best

	**Kind of regression**	**Sum of Squares**	**DOF**	**AICC**	**Best regression AIC-Test**	**Best regression F-Test**	**Delta**	**Probability**	**Likelihood**
50 m breaststroke	polynomial	0.037	1	17.53	linear	linear	23.80	6.75 e^-6^	99.99%
FINA finalists	linear	1.27	4	−6.26					
100 m breaststroke	polynomial	0.17	2	9.28	linear	linear	15.80	0.00036	99.96%
FINA finalists	linear	2.53	6	−6.51					
200 m breaststroke	polynomial	0.21	3	−7.73	linear	linear	3.24	0.16	83.5%
FINA finalists	linear	1.45	6	−10.98					
50 m freestyle	polynomial	0.16	2	9.13	linear	linear	20.57	3.41 e^-5^	99.99%
FINA finalists	linear	1.37	6	−11.44					
100 m freestyle	polynomial	0.56	0	−7.19	linear	undetermined	3.61	0.14	85.9%
FINA finalists	linear	1.48	6	−10.81					
200 m freestyle	polynomial	0.60	0	−6.60	linear	undetermined	6.38	0.039	96.1%
FINA finalists	linear	1.13	6	−12.98					

The changes were all linear.

### Interaction between style and sex

Based on the fastest breaststroke and freestyle swimmers, swimming style and sex had a significant interactive effect on swimming speed for both Swiss (F = 15.13, Dfn = 1, DFd = 2156, *p* = 0.0001) and FINA athletes (F = 6.77, Dfn = 1, DFd = 732, *p* = 0.0094) (Table 7). In Swiss swimmers, style accounted for 61.9% of the total variance of race time (F = 5753.7, Dfn = 1, DFd = 732, *p* < 0.0001) and sex for 14.7% of total variance (F = 1365.6, Dfn = 1, DFd = 2156, *p* < 0.0001), while the interaction accounted for 0.16% (F = 15.1, Dfn = 1, DFd = 2156, *p* = 0.0001). In the FINA swimmers, style accounted for 59.5% of the total variance of race time (F = 1691.3, Dfn = 1, DFd = 732, *p* < 0.0001), sex accounted for 14.5% (F = 412.2, Dfn = 1, DFd = 732, *p* < 0.0001), and the interaction accounted for 0.24% (F = 6.8, Dfn = 1, DFd = 732, *p* = 0.009).

**Table 7:** Mean speed ± SD (m/s) of the top breaststroke and freestyle swimmers over 50 m-200 m distances, at FINA and Swiss competitions during the 1994–2011 period

	**Speed of Swiss swimmers (m/s)** *		**Speed of FINA swimmers (m/s)** **	
	**Breaststroke**	**Freestyle**	**Breaststroke**	**Freestyle**
Men	1.50 ± 0.12	1.91 ± 0.15	1.64 ± 0.10	2.06 ± 0.16
Women	1.33 ± 0.09	1.70 ± 0.11	1.47 ± 0.10	1.84 ± 0.13

All main effects of stroke and sex on swimming speed were highly significant (2-way ANOVA, *p* < 0.0001). A significant interactive effect of swim style x sex is indicated by *(*p* = 0.0001) and **(*p* = 0.009).

## Discussion

The present study examined temporal changes in breaststroke swimming speed for top Swiss and FINA finalists and compared to freestyle swimming speed. The results showed that (*i*) swimming speed increased for both national and international swimmers during the 1994–2011 period for both women and men with the exception for male FINA swimmers in 50 m breaststroke, (*ii*) the sex difference in swimming speed did not change significantly over time except for Swiss freestyle swimmers and (*iii*) the sex-related difference in swimming speed consistently decreased with increasing race distance from 50 m to 200 m for both freestyle and breaststroke.

### Temporal changes in breaststroke and freestyle swimming speed

The increased swimming speed of women and men in freestyle and breaststroke swimming during the 1994–2011 period as well as the new world records during the 2012 Olympic Games [8] are partly attributable to technological advances. Deeper deck-level pools, more effective anti-wave lane ropes, and improved swimming suits reducing drag, improving buoyancy, and enhancing body compression, contributed to the enhanced swimming performance [3,25]. Full-body, polyurethane, technical swimsuits were most probably an important contributor to the unprecedented run of broken records from 1990 to 2009 [26]. A full-body suit improves performance by 3.2 ± 2.4% and reduces drag by 6.2 ± 7.9% for distances from 25 m to 800 m leading to a reduction of energy costs [27]. Indeed, FINA’s release of new rules in 2010 limiting the types of technical swimsuits that could be worn by athletes was followed by a downward trend in performance [26]. However, since 2010, there were still world records in 50 m pools achieved. Especially, world records in breaststroke swimming were improved. For women, Katie Ledecky improved the world records in 800 m and 1,500 m freestyle in 2013, Missy Frankling in 2012 for 200 m backstroke, Ruta Meilutyte in 2013 for 50 m and 100 m breaststroke and Rikke Møller Pederson for 200 m breaststroke. For butterfly, Dana Vollmer improved the world record for 100 m in 2012. For men, Sun Yang improved the 1,500 m freestyle world record in 2012, Cameron van der Burgh in 2012 the world record in 100 m breaststroke, Akihiro Yamaguchi also in 2012 for 200 m breaststroke and Ryan Lochte in 2011 for 200 m individual medley [8].

In addition to technological advances, swimming speed during the 1994–2011 period could have been affected by changes in anthropometric and physiological characteristics [28], improvements in training [29], competition psychology [30,31], and sports nutrition [32], as well as increased access to the sport by a larger number of athletes [4]. A study of 6–17 year-old children during 20^th^ century found an increase of 1–2 cm in body height and 0.5-1.5 kg in body weight per decade [33], a trend which could have led to the improved performances observed in our study. Charles and Bejan [34] observed that the mean body height of champion swimmers in 100 m freestyle increased by 11.4 cm since 1912, and predicted that the fastest athletes would become heavier and taller in future. If available, anthropometric data for Swiss and FINA competitors during the 1994–2011 period could be used to test these assumptions. Due to the linear increase in swimming speed for both national and international swimmers for both breaststroke and freestyle, we may assume that the limits of performance have not reached yet a limit with the exception of the 50 m breaststroke in FINA finalists where the limit of performance may be been reached. However, the period of 1994–2011 might be too short to define whether the change was really linear. Nevill *et al.*[3] investigated the changes in swimming speeds in 100 m, 200 m, and 400 m freestyle world records from 1957 to 2006 and found that a flattened ‘S-shaped curve’ logistic curve best described the changes.

### Sex differences in breaststroke and freestyle swimming

Results of previous studies reporting that the difference between men and women’s world freestyle records remained stable during the past 60 years [3,4,26], were confirmed by results of the present study showing that sex-related differences in breaststroke - and in freestyle swimming by FINA athletes - remained relatively constant over time. The temporal increase in the sex-related difference in freestyle performance by Swiss swimmers is difficult to explain. Different levels of freestyle swimming performance by the variations of velocity, stroke rate, and especially stroke length were found in 100 m freestyle for male swimmers of differing skills (*i.e.* national versus international level) [35]. Additionally, international swimmers are able to maintain a higher energetic and biomechanical capacity than national swimmers [15] and high-speed swimmers have a higher and more stable stroke length and index of coordination than low-speed swimmers [36].

Sex-related differences in performance are largely explained by sex-specific differences in body dimensions, swimming speed, buoyancy, stroke mechanics, stroke length, starts and turn time, basic and specific endurance, anaerobic power and capacity, muscle power, and flexibility [3,37-39]. Young male swimmers have a higher speed fluctuation, active drag, power needed to overcome and technique drag index than young female swimmers [40]. Male freestyle swimmers have also a greater stroke length than female swimmers [36]. Male athletes exhibit greater left ventricular end-diastolic volume than female athletes, resulting in higher stroke volume at rest and during exercise, and higher cardiac output in absolute and relative terms [41]. Men also have 5-10% higher haemoglobin content, which increases oxygen carrying capacity at sub-maximal oxygen uptake levels. These factors lead to greater peak aerobic power, accounting for sex-related differences in the contribution of aerobic energy to exercise [42,43], a factor that is particularly relevant to longer race distances (*i.e.* 100 m-200 m). It has also been reported that pacing strategy differed between women and men [44]. Men applied a positive pacing strategy whereas women applied a negative pacing strategy in 200 m and 400 m medley between 2000 and 2011 for international races [44]. However, it is unlikely that these factors changed significantly during 1994–2011, especially as the change would have had to be greater in Swiss men than in Swiss women to explain the observed results. The gains in swimming speed during the early 21^st^ century following the introduction of new technological swim suits were greater for men than for women in freestyle swimming, while the gains in breaststroke was similar for the two sexes [45]. Although these technological effects might explain the increase in the sex-related difference in Swiss freestyle swimmers, the explanation is countered by the lack of a concomitant increase in FINA swimmers. Differential access to competitive swimming and/or improved training concepts might partly explain the increasing sex-related difference in Swiss freestyle swimming. However, participation in Swiss swimming competition showed a steady annual increase of 3% by both sexes, during 1994–2011 [46].

The stable sex difference at international level is in agreement with previous investigations [4,6,26]. In this highly competitive and demanding environment both men and women have the same access to state-of-the-art training and equipment. It remains to be explored, why at national level men showed a higher increase in swim speed compared to women. Although the sex difference in sport is closing, it remains due to biological differences affecting performance. However, the sex difference is also influenced by reduced opportunity and socio-political factors that influence full female participation across a range of sports around the world [47].

There is also indirect evidence that the sex-related difference might be different in breaststroke compared to freestyle swimming. Active drag coefficient (C_d_) as a measure of technique and performance was lower for faster swimmers [48]. Havriluk found that C_d_ was similar for men and women in freestyle swimming, but that women had a significantly lower C_d_ than men in breaststroke [49]. Therefore, the greater power of men, which allows them to outperform women in freestyle swimming, might not provide such a great advantage in breaststroke.

### Sex difference in swimming performance decreased with increasing distance

The results of the present study showed a decrease in sex-related difference with increasing distance for both breaststroke and freestyle swimming. These results confirm the conclusions of Tanaka and Seals [38] reporting a decrease in the sex-related difference in freestyle performance with increasing race distance from 50 m to 1,500 m. The decrease was attributed to the fact that women were swimming more efficiently than men, and so show relative improvement in performance as distance increases. The more efficient swimming of women is due to their greater fat percentage, shorter legs, and smaller body density, resulting in a more horizontal and streamlined position and smaller body size, which reduces body drag [50,51].

Differences in upper body power might also play a role in the decreasing sex-related difference with increasing race distance [52]. It has been shown that swimming performance was associated with dry land strength of the upper body such as mean power of lat pull down back in elite male freestyle swimmers [53]. Freestyle swimming performance was positively correlated with upper body power in races from 50 m to 400 m [52], but the correlation weakened with increasing race distance [52]. Thus, the greater muscle power of men might become less important with increasing swim distance [54].

It has been assumed that women would be able to outrun men in ultra-marathon running and it was suggested that the sex difference in running would disappear with increasing running distance particularly in distances longer than the marathon [55]. However, a very recent study investigating the sex differences in ultra-marathon running from 50 km to 1,000 km showed that the sex differences in running speeds decreased non-linearly in 50 km and 100 km but remained unchanged in 200 km and 1,000 km [56]. Furthermore, the sex differences in running speeds showed no change with increasing length of the race distance [56]. The findings suggested that it would very unlikely that women will ever outrun men in ultra-marathons held from 50 km to 100 km [56]. For ultra-swimming, however, very recent studies showed that elite female open-water ultra-distance swimmers improved in 10 km but impaired in 25 km leading to a linear decrease in sex difference in 10 km and a linear increase in sex difference in 25 km [57]. The linear changes in sex differences suggest that women will improve in the near future in 10 km, but not in 25 km [57]. However, women might be able to beat men in longer ultra-distances. In the 36 km 'Maratona del Golfo Capri-Napoli' race held from 1954 to 2013, the sex difference in performance decreased linearly from ~38.2% in 1963 to ~6.0% in 2013 [58]. The linear change in both race times and sex differences suggested that women might be able to achieve men's performance or even to outperform men in the near future in open-water ultra-distance swimming. Indeed, in the 46 km 'Manhattan Island Marathon Swim' held in water temperatures <20°C held between 1983 and 2013, the best women were ~12-14% faster than the best men [59]. Most probably the low water temperatures and the higher body fat enable women to beat men in ultra-distance swimming in cold water.

### Limitations and implications for future research

This study has three major limitations. First, we investigated the ten fastest swimmers competing at national level and the eight finalists in the World Championships. The mean of eight swimmers might give a different result compared to the mean of ten swimmers. The 10^th^ fastest woman is not comparable, in terms of level and performance, to the 10^th^ fastest man; this might have influenced the sex difference. Future studies might also normalize the performance (*i.e.* percent of the best performance of each year) which could actually show a better improvement in women’s performance. Second, we used swimming speed as variable to express swimming performance. However, in swimmers, performance could also be compared using FINA points [23]. The ‘FINA Points Table’ allows comparisons of results among different events. The FINA Points Table assigns point values to swimming performances, more points for world class performances typically 1000 or more and fewer points for slower performances [23]. For future studies, performance could be better compared using the ‘FINA Points Table’. Future studies should investigate temporal trends in other swimming styles to determine whether temporal improvement is a generalized phenomenon. Potentially influential factors such as anthropometric characteristics, training, motivation, and nutrition should be included to elucidate the mechanisms underlying temporal changes in athletic performance [16]. Third, we used a time frame of 18 years (1994 to 2011) and investigated the changes across years using linear and non-linear regression analyses. Interestingly, we found only linear changes for both swimming speed and sex difference. In contrast, Nevill *et al.*[3] and Stanula *et al.*[9] reported non-linear changes in freestyle swimming speeds. However, Nevill *et al.*[3] investigated a time period of 50 years (1957–2006) and Stanula *et al.*[9] of 113 years (1986–2008). The shorter time frame in our study most probably explains why we found linear trends and the other authors [3,9] in contrast non-linear trends. The investigation of longer time frames for breaststroke swimmers might show a non-linear trend in both swimming speed and sex difference in swimming speed.

## Conclusion

The results of the present study showed that swimming performance in athletes competing at national and international level improved in freestyle and breaststroke during the 1994–2011 period. The improvement might be related to technological advances, changes in anthropometric and physiological characteristics, improved training methods and competition psychology, improved sports nutrition, and/or increased access to the sport by a larger number of athletes. The sex-related difference in swimming performance did not change significantly over time in breaststroke, or in freestyle swimming by FINA competitors, but increased significantly in Swiss freestyle swimmers. Finally, the sex-related difference in performance declined with increasing race distance in both swim styles.

## Competing interests

The authors declare that they have no competing interests.

## Authors’ contributions

All authors have been involved in collecting data, writing, drafting and revising the manuscript. MW interpreted the data, drafted and revised the manuscript. CAR carried out the data collection, statistical analysis and interpretation. TR participated in its design and revised the manuscript critically for important intellectual content. RL participated in designing and coordinating the study and revised the manuscript critically. BK conceived, designed, coordinated the study and revised the manuscript. All authors read and approved the final manuscript.

## Pre-publication history

The pre-publication history for this paper can be accessed here:

http://www.biomedcentral.com/2052-1847/6/18/prepub
